# Rictor ablation in BMSCs inhibits bone metastasis of TM40D cells by attenuating osteolytic destruction and CAF formation

**DOI:** 10.7150/ijbs.37241

**Published:** 2019-09-07

**Authors:** Zibo Liu, Hui Wang, Jialing He, Xiaoqin Yuan, Weiwei Sun

**Affiliations:** Department of Anatomy, Histology and Embryology, Laboratory of Reproductive Medicine, Research Center for Bone and Stem Cells, Nanjing Medical University, Nanjing, China

**Keywords:** BMSCs, mTOR complex 2, breast cancer bone metastasis, osteolytic bone destruction, CAFs

## Abstract

The mTOR complex 2 (mTORC2) is recognized as a promising target for breast cancer treatment. As mTORC2-specific inhibitors do not yet exist, studies into the role of mTORC2 in cancer are performed by deleting Rictor or by RNAi-mediated Rictor silencing. The purpose of this study was to explore the effects of Rictor ablation in bone mesenchymal stromal cells (BMSCs) on bone metastasis of breast cancer. First, female mice with the genotype of Prx1-Cre;Rictor^f/f^ (hereafter RiCKO) or Rictor^f/f^ (as control) were injected intratibially with cells of the breast cancer cell line (TM40D) at 4 months of age. Three weeks later, osteolytic bone destruction was detected in metastatic legs by X-ray and micro-CT. We found that Rictor ablation in BMSCs inhibited TM40D-induced osteolytic bone destruction and resulted in greater bone volume maintenance *in vivo*. Lower CTX-I serum level, a decreased number of TRAP+ osteoclasts and lower Cathepsin-K expression observed at the tumor-bone interface indicated that osteoclastogenesis was inhibited in RiCKO mice. Additionally, co-culture experiments confirmed that Rictor deletion in BMSCs diminished osteoclast differentiation partly via down regulation of RANKL expression. Furthermore, Rictor deficiency was found to reduce the transition of BMSCs to CAFs coupled with decreased secretion of cytokines (IL-6, RANKL, TGFβ), which resulted in lower chemotaxis and less proliferation in TM40D cells. These results suggest that Rictor ablation in BMSCs plays dual roles in breast cancer bone metastasis: (1) repression of osteolytic bone destruction; (2) inhibition of tumor growth.

## Introduction

Bone is the most common site for breast cancer metastasis 1, 2. Breast cancers that detach from the primary tumor can enter into the circulation, settle in the bone marrow and cause osteolytic bone destruction, leading to a series of complications including bone pain, fracture and hypercalcemia, resulting in serious effects on a patient's quality of life which can even be life threatening^3^. Osteoclasts in the marrow cavity are responsible for osteolytic bone destruction 4. In the marrow microenvironment, cancer cells can directly communicate with pre-osteoclasts to induce osteoclastogenesis or indirectly promote osteoclast differentiation by increasing production of receptor activator of NF-κB ligand (RANKL) 4-7. The disruption of the balance between RANKL and osteoprotegerin (OPG) regulates the course of osteolysis 3, 8. Up to now, bone metastatic disease remains a clinical challenge in breast cancer treatment, as therapeutic managements of breast cancer emphasize controlling primary tumor growth which is not very effective in preventing bone metastasis or metastatic bone disease 1, 9.

The mTORC2, a functional complex of mechanistic target of rapamycin (mTOR) together with the components mTOR, mLST8, Rictor, mSIN1, and Protor1/2, is regarded as apromising target for breast cancer therapy 10-13. Rictor is critical for mTORC2 kinase activity, which controls cell survival, polarity, and cytoskeletal dynamicsthat drive planar cell motility, a key step in metastasis 14, 15. The mTORC2 is reported to be pivotal for motility and invasion of the normal mammary epithelium during branching morphogenesis^16^.Loss of Rictor decreases tumor cell survival and blocks metastasis in ways depending on the Akt, Tiam-1 and Rac1 signaling cascade^11^, ^17^. mTORC2 inhibition also reduces cell motility and survival in cultured human breast cancer cell lines^10^, ^18^, ^19^.Clinical studies have shown that invasive breast carcinomas (IBCs)express more Rictor compared with normal breast epithelium, which suggeststhat Rictor level is correlated with higher grade (grade II/III) breast tumors^17^. Moreover, gains in *RICTOR* gene copy number are associated with decreased overall survival in patients with IBC 17. These preclinical and clinical studies suggest that targeted inhibition of mTORC2 is key for breast cancer therapy. As mTORC2-specific inhibitors do not yet exist, studies into the role of mTORC2 in cancer therapy are circumscribed by deleting Rictor or by RNAi-mediated Rictor silencing 13. Research into the function and regulation of mTORC2 in breast cancers are just getting started, and the comprehensive role of mTORC2 in breast tumor treatment needs further exploration.

BMSCs are recognized to play a critical role during cancer metastasis in the bone marrow microenvironment 20, 21. They are recruited to metastatic sites and secrete factors such as interleukin-6 (IL-6), interleukin-10 (IL-10), interleukin-8 (IL-8), and vascular endothelial growth factor (VEGF), to create a suitable microenvironment for tumor cell seeding and growth 21. BMSCs triggered by cancer cells can also transform into cancer-associated fibroblasts (CAFs). CAFs derived from BMSCs contribute to bone metastasis of cancer by secreting growth factors, modifying the extracellular matrix, supporting angiogenesis, and suppressing anti-tumor immune responses 5, 22. Otherwise, BMSCs are capable of differentiation into osteoblasts, expressing RANKL, M-CSF and OPG to induce differentiation of osteoclasts, while simultaneously influencing bone formation and resorption 23. These findings suggest that BMSCs play multiple roles in the bone metastatic process: BMSCs (1) influence the steady state secretion of cytokines in the marrow microenvironment; (2) affect skeletal tumor progression, and (3) maintain bone homeostasis. mTORC2 is implicated in bone metabolism^24^. mTORC2 signaling promotes osteoclastogenesis by modulating the expression of RANKL. We and others have confirmed that mTORC2 deficiency in BMSCs suppresses osteoclastogenesis and decreases bone resorption in bone marrow by reducing expression of RANKL 24-26. Due to the combination of the effects of mTORC2 and BMSCs on tumor cells and bone turnover aforementioned, it is assumed that mTORC2deficiency in BMSCs has dual effects on anti-tumor progression coupled with bone metabolism in the marrow cavity.

In the present study, we found that Rictor ablation in BMSCs inhibited TM40D-induced osteolytic bone destruction and maintained greater bone volume. Furthermore, Rictor deficiency was found to inhibit the transition of BMSCs to CAFs along with decreased secretion of cytokines. For the first time, our results revealed that targeting mTORC2 could act on BMSCs to restrain skeletal tumor progression and reduce bone destruction. This study enriches the present understanding of mTORC2 and provides justification for developing inhibitors specifically targeting mTORC2 in breast cancer treatment.

## Materials and methods

### Animals

Prx1-Cre mice and Rictor^flox/flox^ (hereafter Rictor^f/f^ ) mice were kindly provided by Dr. Fanxin Long (Washington University in St. Louis, St Louis, MO, USA). Mice with the genotype of Prx1-Cre;Rictor^f/f^ (hereafter RiCKO) were produced as previously described^26^. The genotype of the mice was confirmed by PCR using mouse tail samples. Rictor^f/f^ littermates were used as control animals in all experiments. Nine pairs of 4-month-old RiCKO and Rictor^f/f^ littermates were used in this study. The use of animals in this study was approved by the Institutional Animal Care and Use Committee of Nanjing Medical University (Approval NO.IACUC-1601205).

### Cell line and cell culture

The mammary tumor cell line TM40D was cultured in DMEM (HyClone, Logan, UT, USA), supplemented with 10% fetal bovine serum (v/v) (FBS, HyClone), 100 IU/mL penicillin and 100 mg/mL streptomycin (HyClone) at 37°C.

### Intratibial implantation models

Mice were intratibially injected withTM40D cells as previously described 2. A TM40D cell suspension was slowly injected into the left tibia using a 26G needle to generate bone metastases. Mice were finally sacrificed by cervical dislocation after inhaled anesthesia with ether at 3 weeks post injection. The metastatic legs were explored by imaging, embedded in paraffin after decalcification and finally sliced into 5-μm sections for histological analysis.

### Skeletal radiography and micro-CT analysis

Metastatic tibias were dissected free of soft tissue. X-ray imaging was performed using a Faxitron model 805 (Faxitron Contact, Faxitron, Hennef, Germany) radiographic inspection system (22-kV voltage and 4-min exposure time). Micro-computed tomography (µCT) was performed using a SkyScan 1072 scanner and analysis software (SkyScan, Antwerp, Belgium), with voxel size of 10.5 μm. Analyses of cortical bone parameters were performed on 50-μCT slices (0.8 mm total) at the mid-point of the shaft of the tibia; trabecular parameters were assessed on 120μCT slices (1.6 mm total) immediately below the proximal growth plate of the tibia. Two-dimensional images were used to generate three-dimensional renderings using 3D Creator software supplied with the instrument.

### Histological and immunohistochemical analysis

Paraffin-embedded tissues were cut into5-μm thick sections for histological analysis. For total collagen staining, sections were exposed to 1% Sirius red (Direct red) in saturated picric acid for 1 h, then washed with water thoroughly to remove non-specific staining and counterstained with hematoxylin. Osteoclasts at the tumor-bone interface in bone sections were detected by tartrate-resistant acid phosphatase (TRAP) staining (TRAP staining solution: 25 mg Naphthol AS-MX phosphate and 5 mg Fast Garnet GBC dissolved in 50 mM sodium acetate, 40 mM potassium sodium tartrate buffer)^27^.

Bone sections were deparaffinized, treated with 0.01M sodium citrate buffer by heating at sub-boiling temperature for 15 minutes and processed for immunohistochemical and immunofluorescence staining. The staining process was performed with antibodies against Cathepsin-K (1:100 dilution, Arigo Biolaboratories, Hsinchu City, Taiwan), PCNA (1:100 dilution, Abcam, Cambridge, UK), α-SMA(1:100 dilution, Abcam), and E-cadherin(1:100 dilution, Abcam).The positive area normalized to bone surface was determined using Image J.

### Culture of BMSCs and osteoclastogenesis assay

BMSCs were isolated from tibias and femora of 4-month-old mice as described previously 26. Briefly, bone marrow cells were seeded into 100 mm tissue culture dishes in α-MEM (Gibco/Life Technologies, Carlsbad, CA, USA) containing 10% FBS and 1% penicillin/streptomycin. After 72 hours, the non-adherent cells were removed. On the seventh day, the cells were trypsinized for subsequent experiments.

Tumor-conditioned medium (TCM) was collected from TM40D cells grown in DMEM supplemented with 10% FBS and 1% penicillin/streptomycin for 24h. Bone marrow mononuclear cells (BMMCs) were prepared as previously described 26. Briefly, bone marrow was extracted from bilateral femora and tibiae of 4-month-old Rictor^f/f^ mice and cultured on Petri dishes in α-MEM (Gibco) containing 10% FBS and 1:10 CMG (conditioned medium containing recombinant M-CSF). Cells were cultured at 37°C in 5% CO_2_ for 3 days. Then, BMMCs were washed with PBS, followed by dissociation with 1× trypsin/EDTA (Invitrogen, Carlsbad, CA, USA) in PBS for co-culture with BMSC. Cell suspensions containing1 × 10^4^BMSCsor 3 × 10^4^ BMSCs were co-cultured with 1 × 10^4^BMMCs in TCM mixed with fresh serum-containing DMEM(1:1, v/v) in 48-well tissue culture plates. After co-culture for 7 days, cells were fixed and used for TRAP staining. TRAP-positive cells were regarded as osteoclasts and mature osteoclasts were counted under a microscope according to the number of nuclei (more than four nuclei).

### Western blot analysis

Proteins were extracted from cells and 30 µg samples were fractionated by SDS-PAGE then transferred to nitrocellulose membranes. Membranes were blocked with 5% BSA in TBS-T (TBS with 0.05% Tween 20) and incubated with primary antibodies against p-Akt(473), Akt, p-Foxo3a, Foxo3a, raptor(1:1,000 dilution, Cell Signaling Technology, Danvers, MA, USA); Actin, RANKL, M-CSF, OPG,p16,p19,p27,p21,cdk2,p-NF-κB, NF-κB,p-STAT3, STAT3, α-SMA, and E-cadherin(1:1,000 dilution, Abcam).

### ELISA

Serum CTX-I levels were analyzed using a RatLaps ELISA kit (Immunodiagnostic Systems, Ltd., East Boldon, UK). The animals were fasted for 6 hours before serum collection.

Culture supernatants were collected and the levels of IL-6, TGF-β and RANKL were measured by ELISA, according to the manufacturer's instructions (Abcam).

### Proliferation, apoptosis and migration

Cell proliferation and apoptosis were assessed using 24-well 0.4μm transwell chambers (Merck-Millipore, Darmstadt, Germany). Briefly, 1x10^4^ BMSCs from RiCKO or Rictor^f/f^ mice were first cultured in TCM in a 24‑well plate for 72h to convert into CAFs. Then, 4 × 10^3^ TM40D cells were seeded into inserts placed over the CAFs. The cells were all incubated in DMEM supplemented with 10% FBS and 1% penicillin-streptomycin for 48 h. The membranes were then fixed with 4% paraformaldehyde and used for Edu (Beyotime Institute of Biotechnology, Jiangsu, China) and TUNEL (Roche, Basel, Switzerland) staining according to the manufacturer's instructions.

Cell migration was assessed using 24-well 8μmTranswell chambers (Millipore).Aliquots of 1x10^4^ BMSCs from RiCKO or Rictor^f/f^mice were first cultured in TCM in a 24‑well plate for 72h to convert into CAFs. Then, 1 ×10^5^ TM40D cells were seeded into inserts over the CAFs. The cells were all incubated in DMEM supplemented with 10% FBS and 1% penicillin-streptomycin for 8or 24 h, then the non‑migrated cells were removed from the upper chamber with a cotton swab. The cells that had migrated through the membrane were fixed with 4% paraformaldehyde and stained with 0.1% Crystal Violet. The cells were photographed under the microscope and the cell number was counted from at least five random central fields.

### Statistical analysis

Data are expressed as mean±SEM from at least three separate experiments. Student's t-test was used to determine statistical significance (*P* <0.05). Statistical analyses were performed using GraphPad Prism 7.0 (Graphpad Software Inc., La Jolla, CA, USA).

## Results

### Rictor ablation in BMSCs attenuates TM40D-induced osteolytic lesions* in vivo*

**Figure 1:**
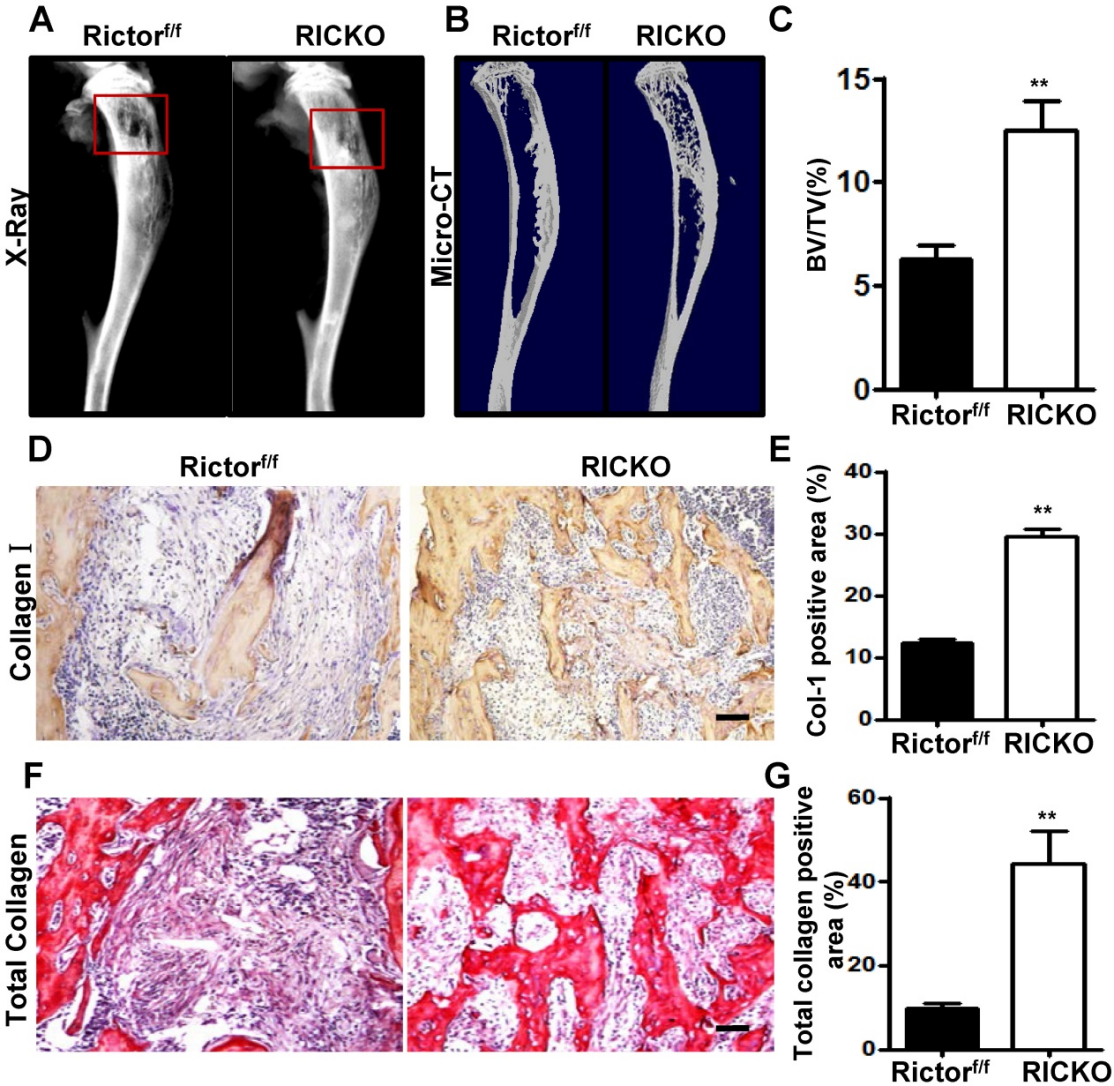
***Rictor ablation in BMSCs attenuates formation of breast cancer cell-induced osteolytic lesion in vivo.***Representative bone destruction in tibiae detected by X-ray (A) and 3-dimensional (3D) micro-CT in longitudinal tibia sections(B).The red wireframes show the metastatic sites. (C) Measurement of bone volume fraction (Bone volume / Total volume). (D) Representative images of collagen-1 staining in metastatic sites. (E) Quantification of collagen-1-positive area relative to section area. (F) Representative images of total collagen staining in metastatic sites. (G) Quantification of total collagen-positive area relative to section area. Scale bar=50 µm. All bar graphs show mean ± SEM. **P <0.01.

To explore the influence of Rictor ablation in BMSCs onTM40D-induced bone destruction, TM40D breast cancer cells were injected intratibially intoPrx1-Cre; Rictor^f/f^(hereafter RiCKO) mice or Rictor^f/f^ mice (as controls), to construct a breast cancer bone metastasis model*.* Three weeks later, X-ray and µCT imaging were used to examine bone metastasis. Bone osteolysis was detected in the mice of both genotypes, which signified that bone metastasis of TM40D was successfully established. Compared with Rictor^f/f^ mice, less bone osteolysis appeared in RiCKO mice as shown by X-Ray (Fig. 1A) and µCT 3-dimensional (3D) images (Fig. 1B). The micro-CT analysis revealed that more bone volume was maintained in RiCKO mice than in control groups (Fig. 1C). In accordance with the radiological data, the immunohistochemical staining of collagen-I and histochemical staining of total collagen confirmed that bone mass was greater in the metastatic sites of RiCKO mice (Fig. 1D,E).These results indicated that Rictor ablation in BMSCs inhibited cancer-induced osteolytic lesions and maintained greater bone volume.

**Figure 2:**
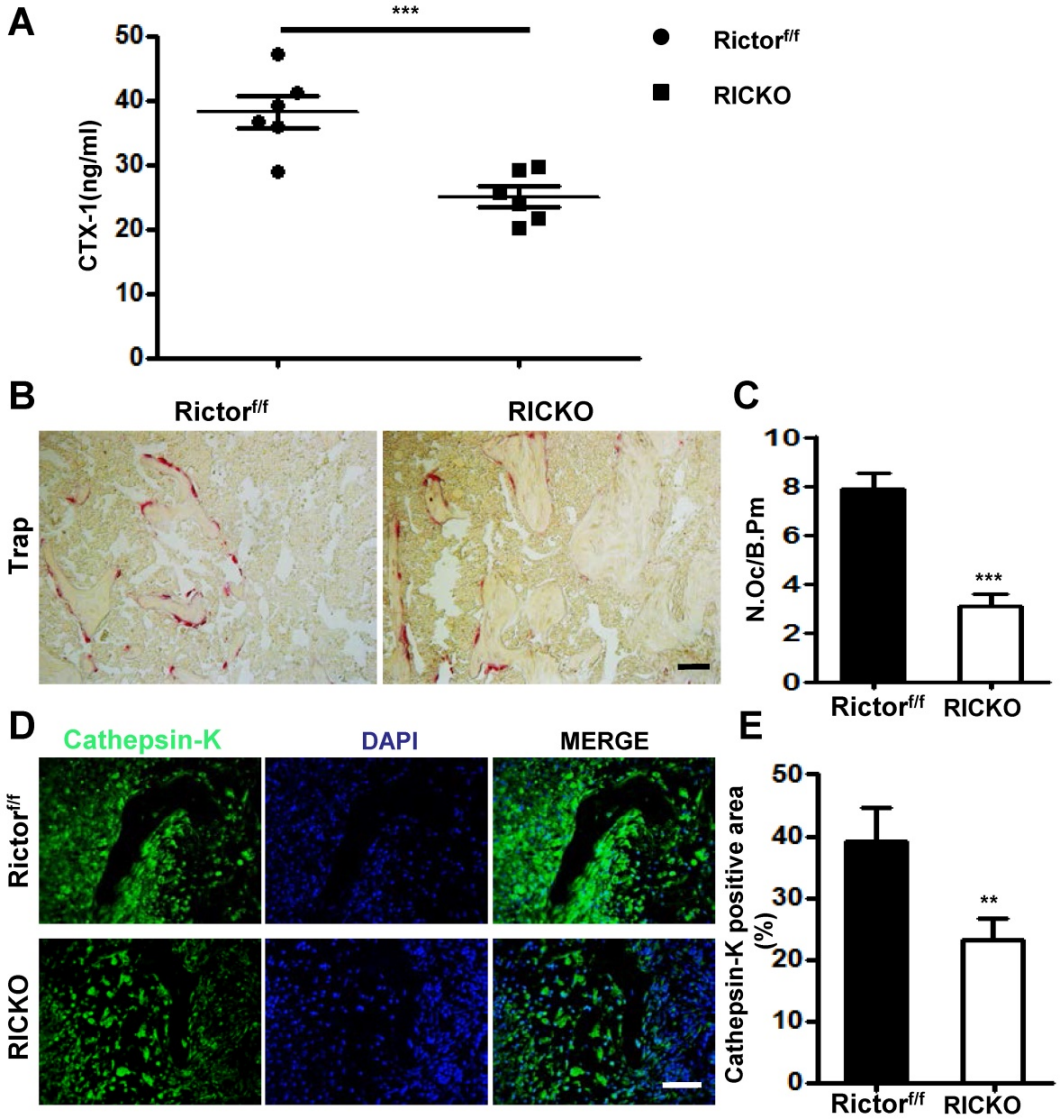
***Rictor ablation in BMSCs decreases osteoclast formation in vivo.***(A) Serum CTX-1 levels. (B) Representative images of TRAP staining in metastatic sites. (C) Quantification of osteoclast number normalized to trabecular bone surface. (D) Representative images of Cathepsin-K staining at the tumor-bone interface. (E) Quantification of Cathepsin-K-positive area relative to section area. Scale bar=50 µm. All bar graphs show mean ± SEM. **P <0.01, ***P< 0.001.

To determine the cellular basis for the reduction in osteolytic lesions found in RiCKO mice, we measured serum levels of CTX- I, a common marker of bone resorption activity. The serum level of CTX-I was significantly lower in RiCKO mice than in their control littermates (Fig. 2A). Consistent with the lower CTX-I levels, TRAP staining of the tumor-bone interface revealed an apparent reduction in the number of TRAP^+^ osteoclasts normalized to bone surface (N. Oc/B. Pm) in the RiCKO mice (Fig. 2B,C). Furthermore, immunofluorescent staining showed decreased expression of Cathepsin-K, a key protease for bone destruction, at the tumor-bone interface in the RiCKO mice compared with the control mice (Fig. 2D,E). These results indicated that osteoclastogenesis is inhibited at the tumor-bone interface in RiCKO mice, resulting in suppression of cancer-induced bone destruction *in vivo*.

### Rictor ablation in BMSCs suppresses TM40D-induced osteoclastogenesis *in vitro*

**Figure 3:**
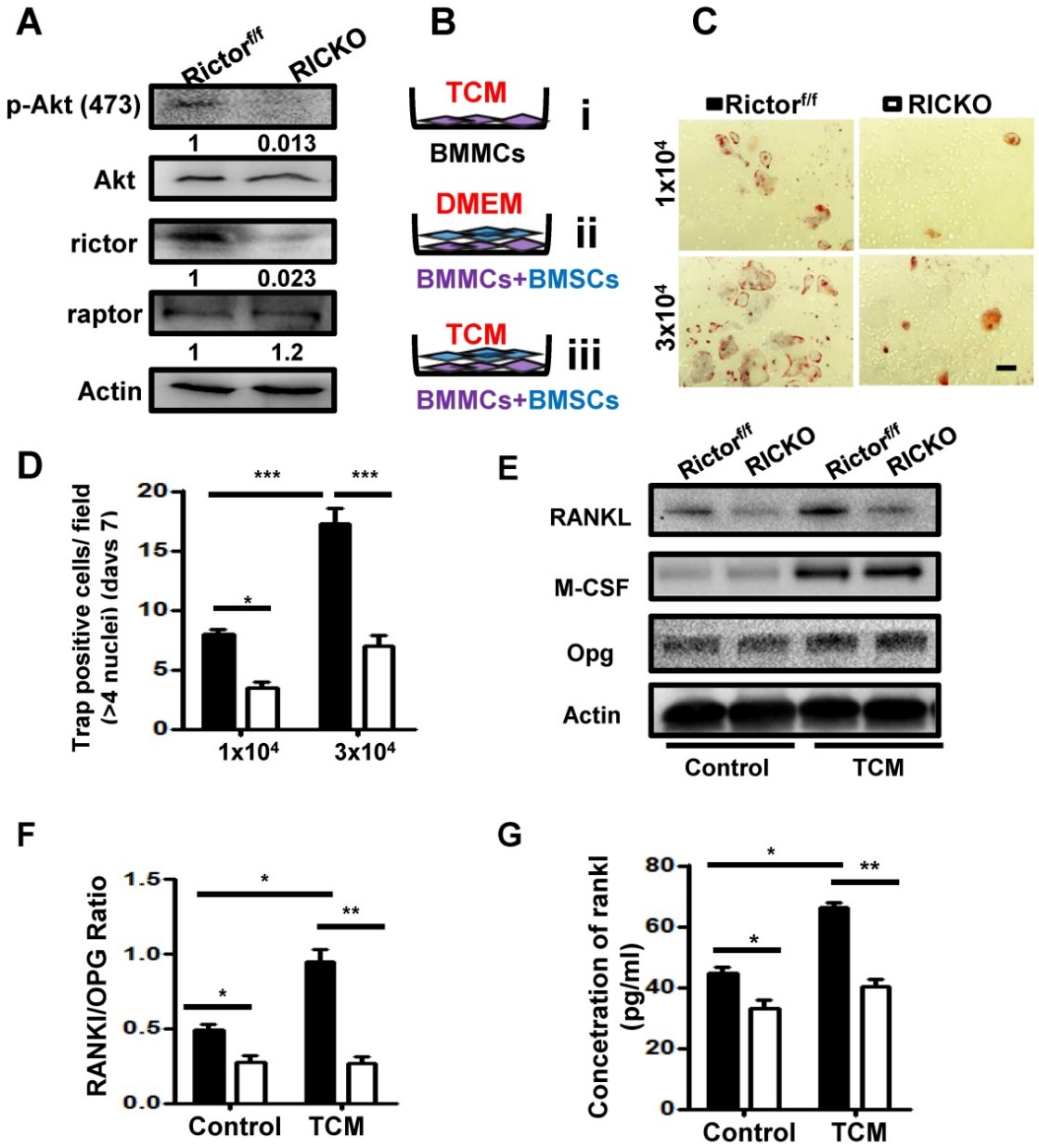
*** Rictor ablation in BMSCs suppresses TM40D induced osteoclastogenesis in vitro.***(A) Western blot analyses of BMSCs isolated from Rictor^f/f^ and RiCKO mice. (B) Schematic diagram of cell culture. (C) Representative TRAP staining of osteoclasts differentiated from macrophages co-cultured with BMSCs in TCM. (D) Quantification of TRAP^+^ cells. (E) Western blot analyses of BMSCs isolated from Rictor^f/f^ and RiCKO mice cultured in control media or TCM.(F) Relative ratio of RANKL to OPG calculated from Western blot. (G) ELISA quantification of RANKL protein level in supernatant of BMSCs after treatment with control or conditioned medium (TCM).Scale bar=50 µm. All bar graphs show mean ± SEM. *P <0.05;**P <0.01; ***P < 0.001.

To further investigate the impact of Rictor-deficient BMSCs on supporting osteoclastogenesis induced by cancer, we performed direct co-culture experiments. BMSCs were extracted from RiCKO or Rictor^f/f^ mice. Western blotting showed that Rictor levels and phosphorylation of AKT at S473, a direct readout of mTORC2 activity, were both markedly reduced in the BMSCs obtained from RiCKO mice (Fig. 3A). Bone marrow mononuclear cells (BMMCs) were then cultured inTCM (Fig. 3B i) or directly co-cultured with3 × 10^4^/well BMSCs obtained from RiCKO or Rictor^f/f^ mice in DMEM without adding RANKL or M-CSF (Fig. 3B ii). Thirteen days later, no TRAP^+^ osteoclasts were detected (data not shown).These results revealed that TCM or 3 × 10^4^/well BMSCs alone could not maintain osteoclastogenesis *in vitro* without RANKL or M-CSF. We then divided the BMMCs into two groups, and each group was directly co-cultured with BMSCs obtained from RiCKO or Rictor^f/f^ mice in TCM (Fig. 3B iii).After 7 days, the number of TRAP^+^ osteoclasts with >4 nuclei was counted. As indicated, BMSCs from both RiCKO and Rictor^f/f^ mice adequately preserved osteoclast differentiation in TCM without RANKL or M-CSF addition (Fig. 3C).

The quantification of TRAP^+^ cells revealed that less osteoclasts differentiation was induced by co-culture with 1 × 10^4^ BMSCs from the RiCKO mice than from littermate controls (*P <0.05). When the number of BMSCs was increased to 3 × 10^4^/well, the number of TRAP^+^ osteoclasts also rose significantly (***P <0.001) (Fig. 3D). These results suggested that BMSCs and TCM are both pivotal for osteoclastogenesis *in vitro* and that Rictor ablation in BMSCs suppresses osteoclastogenesis.

To examine the molecular basis for osteoclast differentiation, we evaluated the levels of several known osteoclastogenic factors including RANKL, OPG and M-CSF expressed in BMSCs cultured in DMEM (as control) or TCM, for 7days (Fig. 3E). As we reported previously, RANKL expression was significantly reduced, whereas expression levels of OPG and M-CSF were not altered in RICKO BMSCs compared with Rictor^f/f^ BMSCs cultured in DMEM. After 7days of TCM treatment, expression of M-CSF increased significantly in both Rictor^f/f^ and RiCKO BMSCs. However, the expression of RANKL did not rise in RiCKO BMSCs as much as in Rictor^f/f^ BMSCs after treatment with TCM. As a result, the ratio of RANKL to OPG (RANKL/OPG) was more pronounced between Rictor^f/f^ BMSCs and RiCKO BMSCs in TCM (Fig. 3F). Consistent with the results of western blotting, the result of ELISA confirmed that the protein level of RANKL was not increased in RiCKO cellular supernatant as much as in Rictor^f/f^ supernatant after treatment with TCM (Fig. 3G).These results demonstrate that Rictor is crucial for TCM-induced RANKL expression in BMSCs, suggesting that reduction of RANKL expression by BMSCs in RiCKO mice is a major mechanism for the decreased osteoclastogenesis induced by cancer cells.

### Loss of Rictor inhibits the transition of BMSCs to cancer associated fibroblasts (CAFs)

**Figure 4:**
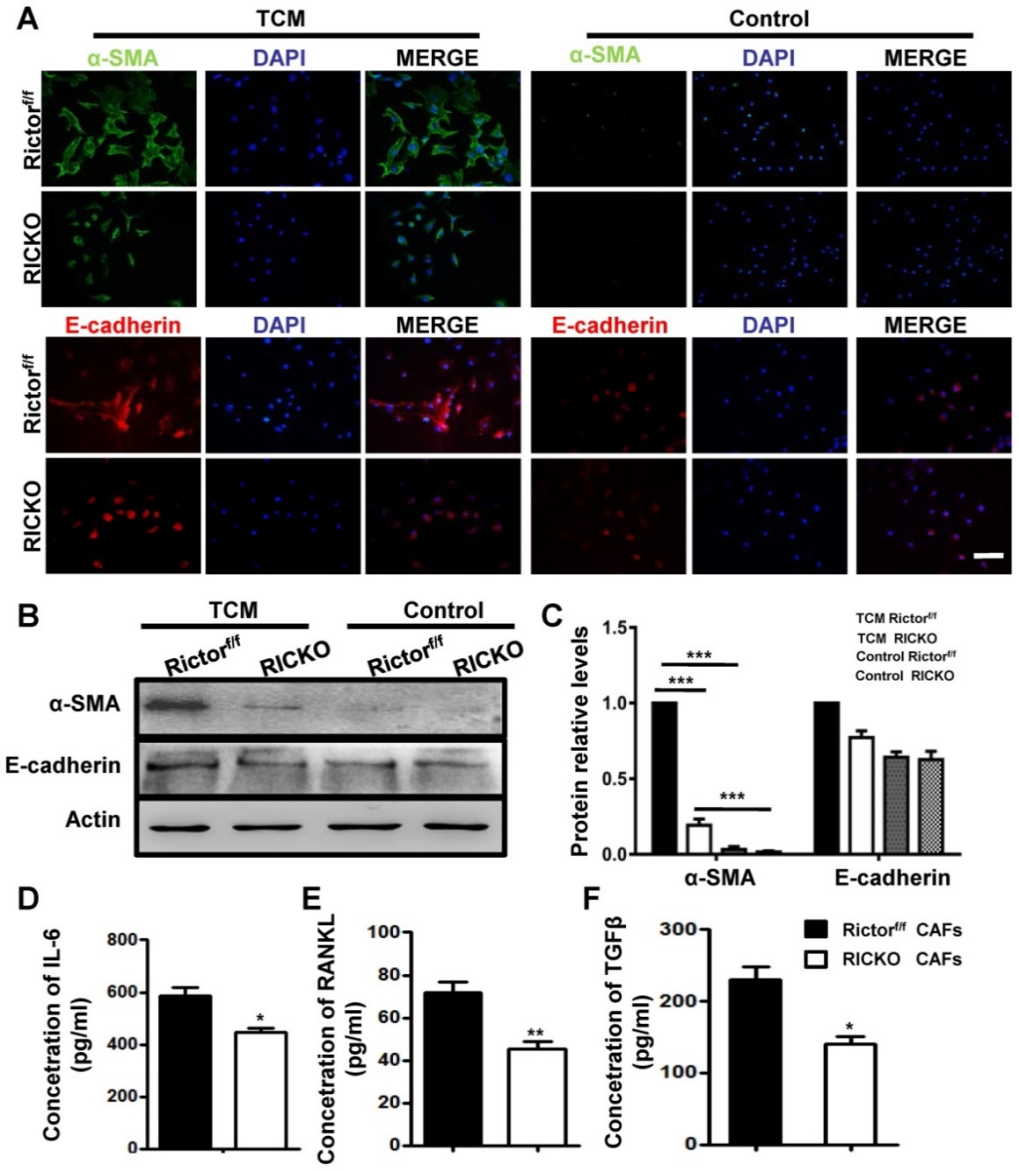
***Loss of Rictor inhibits the transition of BMSCs to Cancer associated fibroblasts (CAFs).***(A) Representative images of α-SMA and E-cadherin staining of BMSCs cultured in TCM or normal medium (for control). (B) Western blot analyses of α-SMA and E-cadherin expression in BMSCs cultured in TCM or normal medium.(C) The protein expression levels are calculated as a ratio to the Actinprotein level and expressed relative to levels in group of TCM Rictor^f/f^. (D-F) Quantitation of cytokine levels by ELISA. Scale bar=100 µm. All bar graphs show mean ± SEM. *P <0.05; **P< 0.01; ***P< 0.001.

As important resident fibroblasts in the bone marrow, BMSCs have been recognized to play a critical role during bone metastasis due to their ability to differentiate into cancer-associated fibroblasts (CAFs) and to secrete cytokines into the tumor microenvironment (TME)^28^. We next investigated the role of Rictor in the transition of BMSCs to CAFs. We collected conditioned medium (TCM) from cultured TM40D cells. BMSCs from Rictor^f/f^ or RiCKO mice were then cultured in TCM or in normal medium (as control) for 72h and the expression of CAF-related markers were evaluated. The biomarkers of CAFs, including smooth muscle actin α (α-SMA) and E-cadherin, were determined using immunofluorescent staining and western blotting (Fig. 4A, B). As shown in Fig. 4C, the biomarkers of CAFs in the TCM group were significantly upregulated compared with the control group, which indicated that TM40D cells might mediate the conversion of BMSCs to CAFs. Furthermore, the expressions of biomarkers increased more in CAFs from Rictor^f/f^ mice (Rictor^f/f^ CAFs) than from RiCKO mice (RiCKO CAFs).

Next we analyzed the cytokines secreted by CAFs using ELISA. The supernatants of CAFs were collected after 48h in culture. We identified three cytokines (IL-6, RANKL, TGFβ) with significantly decreased secretion in the culture medium from RiCKO CAFs, compared to medium from Rictor^f/f^ CAFs (Fig. 4D,E,F). These three cytokines have been reported to play positive roles in promoting cancer cell growth and metastasis 3, 29-31. Taken together, these results suggested that Rictor deficiency inhibited the transition of BMSCs to CAFs as well as decreasing cytokine secretion.

### Less proliferation and chemotaxis of TM40D cells are observed when co-cultured with Rictor-deficient CAFs

**Figure 5:**
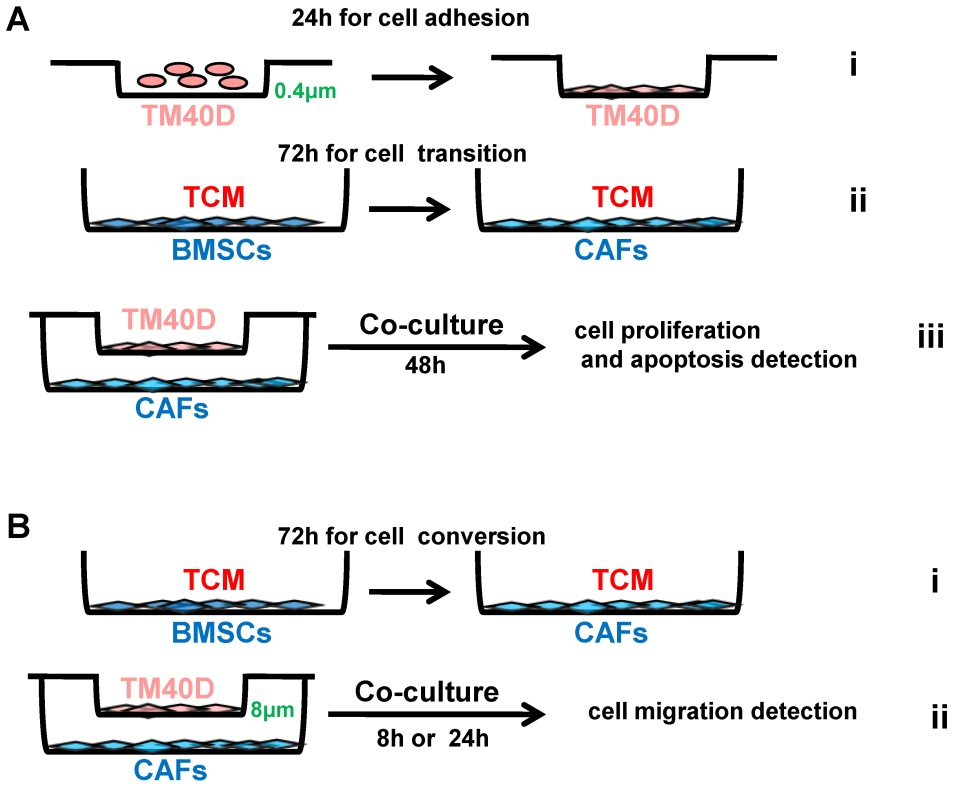
***Schematic diagram of cell co-culture.***Tumor conditioned medium (TCM) was collected from TM40D cells grown in DMEM supplemented with 10% FBS and 1% penicillin/streptomycin for 24h. (A) TM40D cells were plated into 0.4μm transwell inserts and allowed to adhere to their supports for 24h (i); 1 × 10^4^ BMSCs from RiCKO or Rictor^f/f^ mice were cultured in TCM in a 24‑well plate for 72h to change into CAFs (ii); The seeded inserts were placed over the CAFs and co-cultured for 48h (iii). (B) 1 × 10^4^ BMSCs from RiCKO or Rictor^f/f^ mice were cultured in TCM in a 24‑well plate for 72h to convert to CAFs (i); TM40D were plated into 8 μm transwell chambers for co-culture with CAFs for 8 or 24h (ii).

**Figure 6:**
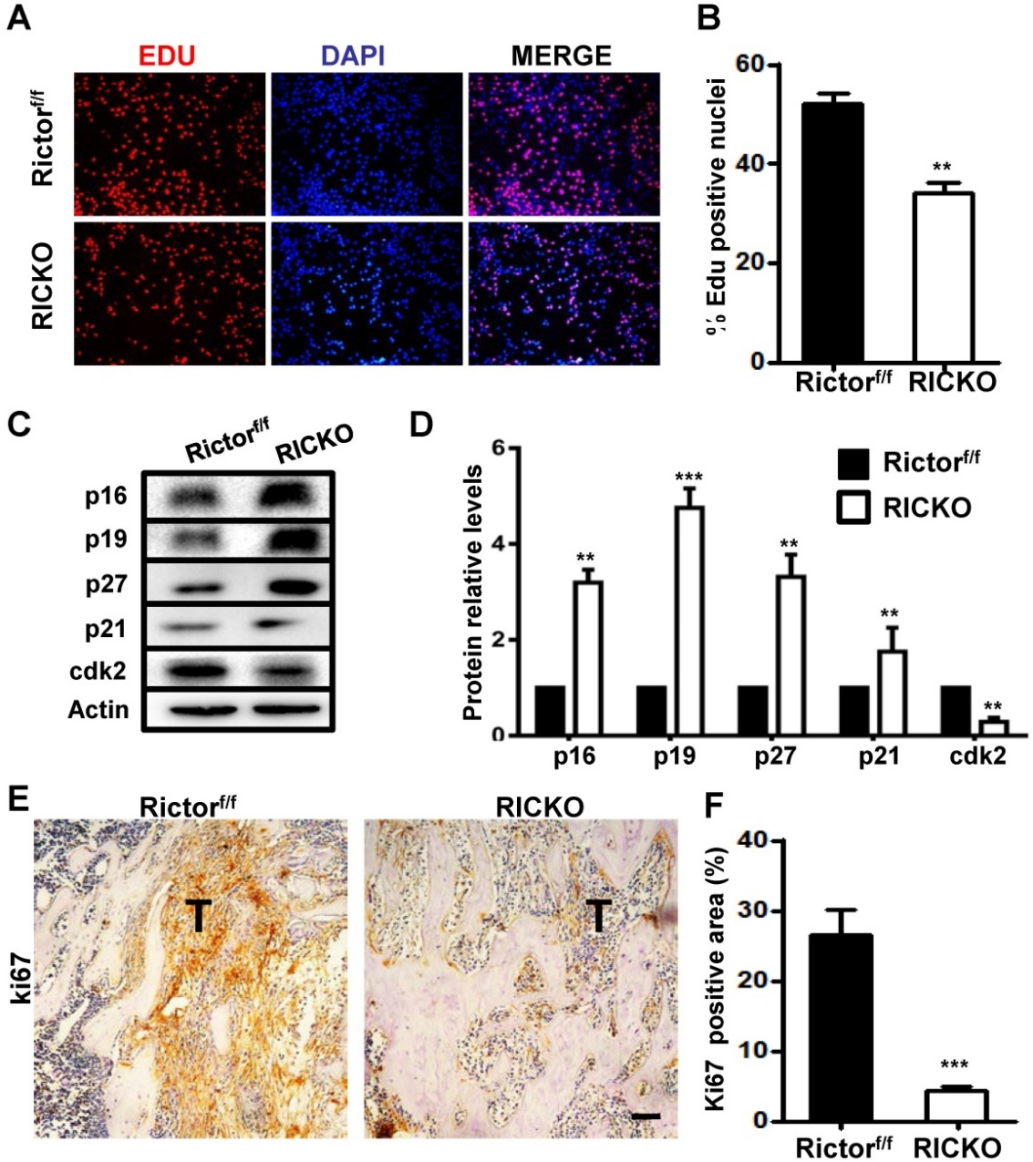
***Rictor deficient CAFs block TM40D cell growth.***(A) Representative images of Edu staining. (B) Quantification of Edu-positive cells relative to total cells. (C) Western blot analyses of cyclin-dependent kinase inhibitors (p16,p19,p21,p27) and cyclin-dependent kinase cdk2. (D) The protein expression levels are calculated as a ratio to the Actin protein level and expressed relative to levels in the Rictor^f/f^ group. (E) Representative images of ki67 staining in metastatic sites *in vivo*. (F) Quantification of the ki67-positive area relative to section area (T means tumor). Scale bar=100 µm. All bar graphs show mean ± SEM. **P <0.01; ***P< 0.001.

To further investigate the impact of CAFs on cancer cell growth and apoptosis, we performed co-culture experiments. TM40D cells were first plated into 0.4μm transwell inserts and allowed to adhere to their supports. After 24h the seeded inserts were placed over RiCKO CAFs or Rictor^f/f^ CAFs to start co-culture. A further 48 h later, we evaluated the proliferation and apoptosis of TM40Dcells (Fig. 5A). Fluorescent staining of Edu showed a decrease in the Edu^+^ proportion of TM40D cells after co-culture with RiCKO CAFs (Fig. 6A, B). The protein expression of cell cycle regulatory proteins was further analyzed in TM40D cells after co-culture. Western blot analysis showed that the cyclin-dependent kinase inhibitors (p16, p19, p21, p27) were up regulated and cyclin-dependent kinase (cdk2) was down-regulated in TM40D cells co-cultured with RiCKO CAFs, which resulted in cell cycle arrest and reduced cell proliferation (Fig. 6C,D). However, there was no significant difference in the apoptosis level of TM40D cells co-cultured with RiCKO CAFs or Rictor^f/f^ CAFs (data not shown).

Next, we further tested the proliferation of tumor cells *in vivo*. We immunohistochemically stained tissue sections for ki67 (Fig. 6E). In accord with results obtained *in vitro*, fewer ki67-positive cells were observed in the tumor site from RiCKO mice compared with control littermates (Fig. 6F). Taken together, these results suggested that Rictor-deficient CAFs suppressed the proliferation of TM40D cells *in vitro* and *in vivo*.

**Figure 7:**
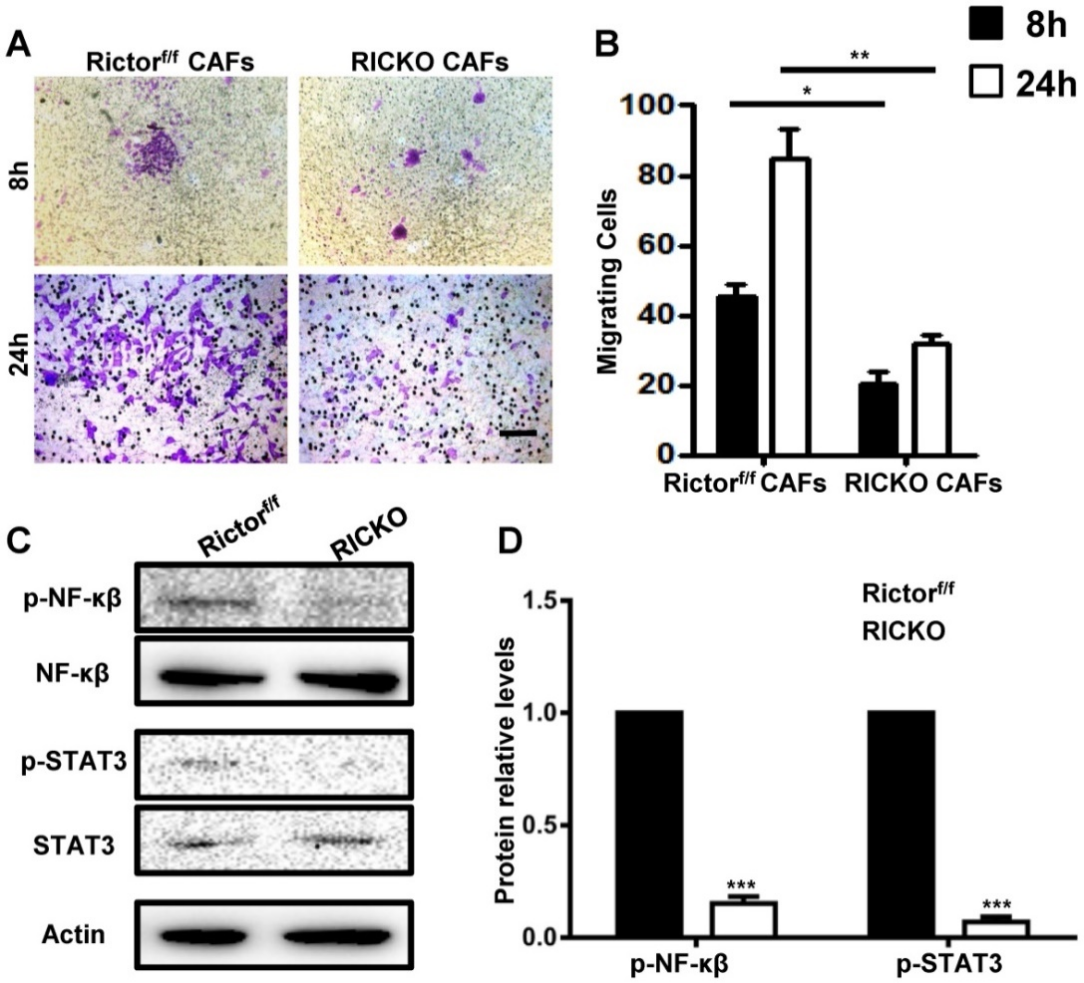
***Rictor deficiency weakens the chemotactic attraction of CAFs to TM40D cells.***(A) Representative images of migratory TM40D cells that were co-cultured with CAFs for 8 or 24h.(B) The calculated number of TM40D cells. (B) Western blot analyses of downstream proteins of IL-6 and RANKL.(C) The protein expression levels of p-NF-κβ and p-STAT3, calculated as a ratio to the NF-κβ and STAT3protein levels respectively, and expressed relative to levels in group of Rictor^f/f^. Scale bar=100 µm. All bar graphs show mean ± SEM. *P <0.05; **P <0.01; ***P< 0.001.

To explore the chemotactic attraction of CAFs to TM40D cells, the cancer cells were incubated with CAFs in 8 μm Transwell chambers (Fig. 5B). After 8 or 24h, the migrated cells were stained with Crystal Violet and stained cells were counted in at least five random high‑power fields (Fig. 7A). The cell counting analysis showed that fewer TM40D cells migrated to RiCKO CAFs compared with those migrating to Rictor^f/f^ CAFs (Fig. 7B). These results revealed that Rictor deficiency weakened the chemotactic attraction of CAFs to TM40D cells.

To further identify whether cytokines secreted by CAFs influence cancer cell growth and chemotactic attraction detected above, we examined the downstream proteins of IL-6 and RANKL in co-cultures with TM40D cells. Consistently, phosphorylated NF-κβ (p-NF-κβ) and STAT3 (p-STAT3) were expressed at lower levels in TM40D cells co-cultured with RiCKO CAFs (Fig. 7C, D). These results suggested that down regulation of the IL-6/STAT3 and RANK-RANKL signal pathways contributed to inhibition of TM40D cell growth and chemotactic attraction.

## Discussion

An active PI3K/Akt/mTOR signaling cascade that drives tumor cell growth, survival, metabolism, and motility has been generally recognized in cancers during recent years 32, 33. The serine/threonine kinase mTOR exists in two structurally- and functionally-distinct complexes, mTORC1 and mTORC2.mTORC1 specifically contains protein of mammalian target of rapamycin (raptor), whereas mTORC2 is uniquely comprised of Rictor, which is rapamycin-insensitive but responsive to growth factor signaling, and functions mainly through AKT activation by phosphorylation of itsS473 site. Rapamycin and its analogs targeting mTORC1have been propelled into preclinical and clinical trials for breast cancer therapy 34-39. However, the effect of single targeting mTORC1 is limited 40, 41. Moreover, several recent studies considered that increased activity of mTORC2, but not of mTORC1, is essential for the development of breast cancers. Amplification of Rictor is correlated with metastasis and therapeutic resistance in triple-negative breast cancer 14, 42. RNAi-mediated knockdown of Rictor inhibits mTORC2 activity in MCF7 and PC3 tumor cells 18. Rictor expression is upregulated by Runt-related transcription factor (Runx2) in invasive breast cancer cells 43. Also, interferon regulatory factor-4 binding protein has been found to directly activate the mTORC2/Akt/forkhead box O (FoxO)-3a axis in invasive human breast cancer cells 44. In the present study, we used a transgenic mouse model to investigate the effects of Rictor ablation in BMSCs on bone metastasis of breast cancer cells. We found that Rictor ablation in BMSCs inhibited TM40D-induced osteolytic bone destruction and maintained greater bone volume by diminishing osteoclast differentiation and RANKL expression. Furthermore, Rictor deficiency was found to deter the transition of BMSCs to CAFs along with decreased secretion of cytokines (IL-6, RANKL, TGFβ), which resulted in lower chemotaxis and less proliferation observed in TM40D cells.

Metastatic breast cancer triggers disruption of bone homeostasis, and ultimately results in formation of osteolytic bone lesions 1, 9.During osteoclastogenesis induced by cancer cells, RANK/RANKL signaling has been confirmed to stimulatethe expression of key transcription factors including: nuclear factor κB (NFκB), activator protein-1 (AP-1), and nuclear factor of activated T-cells cytoplasmic 1(NFATc1)^7^, ^45^. Osteoprotegerin (OPG) is a competitive receptor for RANKL, binding to RANKL and inhibiting RANKL/RANK signaling and negatively regulating osteoclastogenesis. Moreover, increased RANKL/RANK/OPG expression has been shown to be related to breast cancer cell proliferation and migration 3, 46. For the treatment of metastatic breast cancer, RANK/RANKL inhibitors having been suggested as bone-targeted therapy in patients with metastatic bone disease 8, 47. In recent years, mTOR has been recognized to be involved in the anti-apoptotic effects of RANK/ RANKL on osteoclasts 34.

However, application of mTOR inhibitors in cancer therapy is concerning because of their potential toxicity. Moreover, activation of autophagy and negative feedback loops are induced by mTORC1 inhibition. We have investigated whether mTORC2 is also involved in expression of RANKL. We found that the expression of RANKL was inhibited in Rictor-deficient BMSCs, which contributed to reduced osteoclast differentiation from macrophages^26^.In this study, we performed direct co-culture experiments to investigate the impact of Rictor- deficient BMSCs on supporting osteoclastogenesis induced by cancer. We found that Rictor was crucial for TCM-induced RANKL expression by BMSCs.

Cancer-associated fibroblasts (CAFs) play critical roles in proliferation, migration and invasion of several different types of tumor cells 22. As an important component of the bone marrow microenvironment, BMSCs have been reported to possess the ability to change into CAFs, which become more aggressive, contractile and produce pro-tumorigenic cytokines 28, 48. Cytokines such as TGF-α, TGF-β, IL-6, and bFGF in the bone marrow microenvironment lead to activation of BMSCs and their conversion into CAFs. At the same time, these cytokines activate both tumor cells and CAFs in an autocrine-paracrine manner, which results in increased tumor cell proliferation, decreased apoptosis, increased invasion, and promotion of tumor growth 29, 31, 49. Active IL-6/STAT3 (MEK1/2) and bFGF/Erk/Smad3 signaling pathways have been suggested to be involved in the transition of CAFs.

In this paper, to investigate whether the mTORC2 signaling pathway participates in the transition of BMSCs to CAFs, BMSCs from Rictor^f/f^ or RiCKO mice were cultured in TCM or in normal medium. We found that all the biomarkers of CAFs increased less in RiCKO BMSCs than in Rictor^f/f^ BMSCs. Three cytokines (IL-6, RANKL, and TGF-β) displayed significantly decreased secretion in the culture medium from RiCKO CAFs. We then used CAFs converted from RiCKO BMSCs or Rictor^f/f^ BMSCs in co-culture with TM40D cells to investigate the impact of CAFs on cancer cell growth and chemotactic attraction. As we predicted, proliferation was inhibited and chemotactic attraction of CAFs to TM40D cells was suppressed when Rictor was deficient. In addition, the reduced expression of phosphorylated NF-κβ (p-NF-κβ) and STAT3 (p-STAT3) in TM40D cells co-cultured with CAFs converted from RiCKO BMSCs suggested that the down-regulated IL-6/STAT3 and RANK-RANKL signal pathways contribute to inhibition of TM40D cell growth and chemotactic attraction.

In conclusion, the present findings revealed that Rictor ablation in BMSCs inhibitedTM40D-induced osteolytic bone destruction and transition into CAFs. Combining these findings with the role of mTORC2 in tumor growth as previously reported, it may be hypothesized that targeting mTORC2 could not only inhibit tumor development and bone metastasis, but also ease the lytic osseous bone disease caused by bone metastases.
